# Dendrimers: synthesis, applications, and properties

**DOI:** 10.1186/1556-276X-9-247

**Published:** 2014-05-21

**Authors:** Elham Abbasi, Sedigheh Fekri Aval, Abolfazl Akbarzadeh, Morteza Milani, Hamid Tayefi Nasrabadi, Sang Woo Joo, Younes Hanifehpour, Kazem Nejati-Koshki, Roghiyeh Pashaei-Asl

**Affiliations:** 1 Department of Medical Nanotechnology, Faculty of Advanced Medical Sciences, Tabriz University of Medical Sciences, Tabriz 5154853431, Iran; 2 Department of Medical Biotechnology, Faculty of Advanced Medical Sciences, Tabriz University of Medical Sciences, Tabriz 5154853431, Iran; 3 Department of Molecular Medicine, Faculty of Advanced Medical Sciences, Tabriz University of Medical Sciences, Tabriz 5154853431, Iran; 4 School of Mechanical Engineering, Yeungnam University, Gyeongsan 712-749, South Korea

**Keywords:** Dendrimer, Pseudorotaxane, Nanoscale, PAMAM

## Abstract

Dendrimers are nano-sized, radially symmetric molecules with well-defined, homogeneous, and monodisperse structure that has a typically symmetric core, an inner shell, and an outer shell. Their three traditional macromolecular architectural classes are broadly recognized to generate rather polydisperse products of different molecular weights. A variety of dendrimers exist, and each has biological properties such as polyvalency, self-assembling, electrostatic interactions, chemical stability, low cytotoxicity, and solubility. These varied characteristics make dendrimers a good choice in the medical field, and this review covers their diverse applications.

## Review

### Introduction

**Figure 1:**
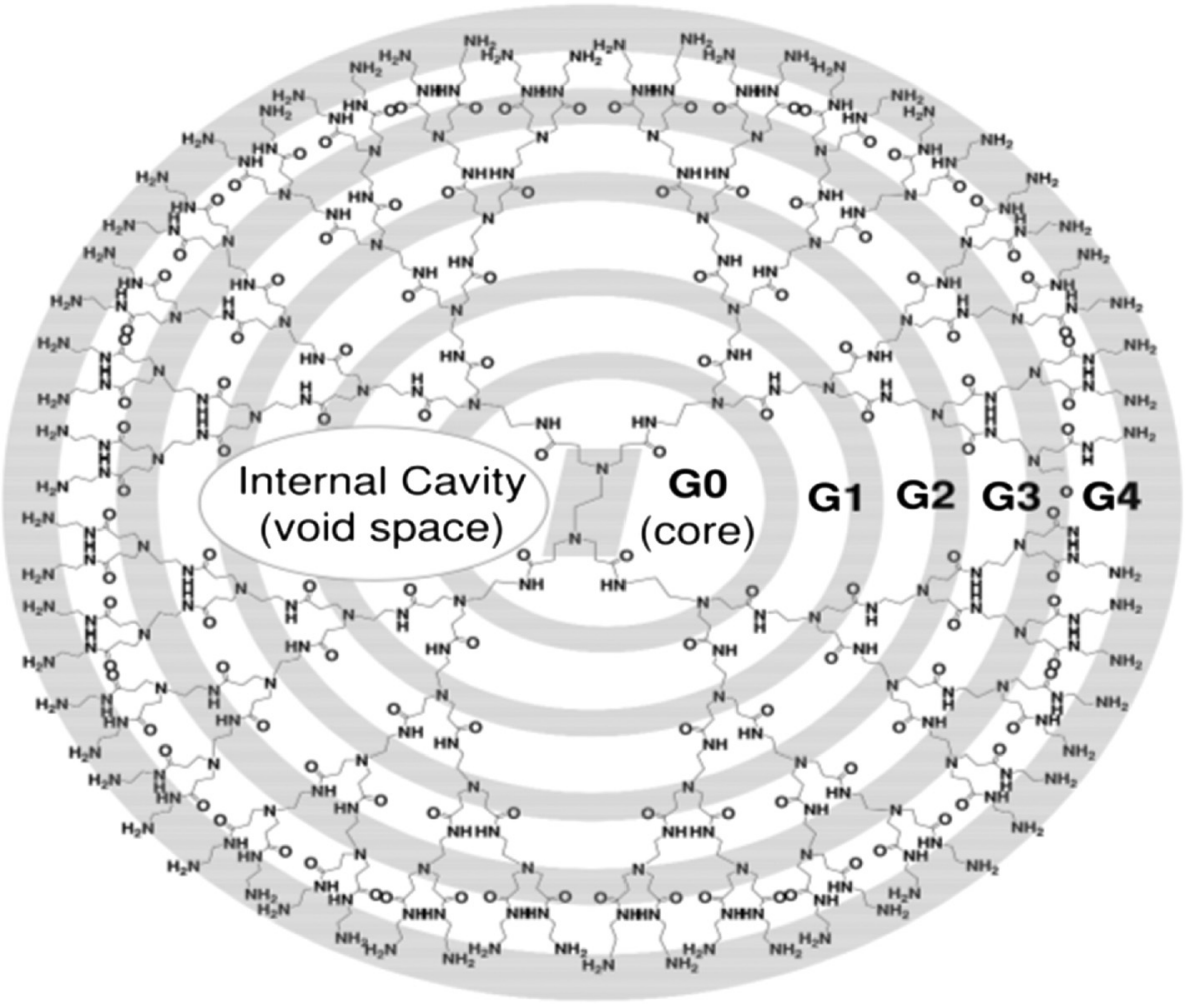
**Schematic representation of a generation G4 dendrimer with 64 amino groups at the periphery.** This dendrimer starts from an ethylene diamine core; the branches or arms were attached by exhaustive Michael addition to methyl acrylate followed by exhaustive aminolysis of the resulting methyl ester using ethylene diamine [20].

Dendrimers are nano-sized, radially symmetric molecules with well-defined, homogeneous, and monodisperse structure consisting of tree-like arms or branches [1]. These hyperbranched molecules were first discovered by Fritz Vogtle in 1978, by Donald Tomalia and co-workers in the early 1980s, and at the same time, but independently by George R. Newkome. The second group called synthesized macromolecules ‘arborols’ means, in Latin, ‘trees’. Dendrimers might also be called ‘cascade molecules’ , but this term is not as much established as ‘dendrimers’ [2-4]. Dendrimers are nearly monodisperse macromolecules that contain symmetric branching units built around a small molecule or a linear polymer core [5-7]. ‘Dendrimer’ is only an architectural motif and not a compound. Polyionic dendrimers do not have a persistent shape and may undergo changes in size, shape, and flexibility as a function of increasing generations [8-10]. Dendrimers are hyperbranched macromolecules with a carefully tailored architecture, the end-groups (i.e., the groups reaching the outer periphery), which can be functionalized, thus modifying their physicochemical or biological properties [11-16]. Dendrimers have gained a broad range of applications in supramolecular chemistry, particularly in host-guest reactions and self-assembly processes. Dendrimers are characterized by special features that make them promising candidates for a lot of applications. Dendrimers are highly defined artificial macromolecules, which are characterized by a combination of a high number of functional groups and a compact molecular structure [17]. The emerging role of dendritic macromolecules for anticancer therapies and diagnostic imaging is remarkable. The advantages of these well-defined materials make them the newest class of macromolecular nano-scale delivery devices [18]. Dendritic macromolecules tend to linearly increase in diameter and adopt a more globular shape with increasing dendrimer generation. Therefore, dendrimers have become an ideal delivery vehicle candidate for explicit study of the effects of polymer size, charge, and composition on biologically relevant properties such as lipid bilayer interactions, cytotoxicity, internalization, blood plasma retention time, biodistribution, and filtration [19] (Figure 1).

### Structure and chemistry

**Figure 2:**
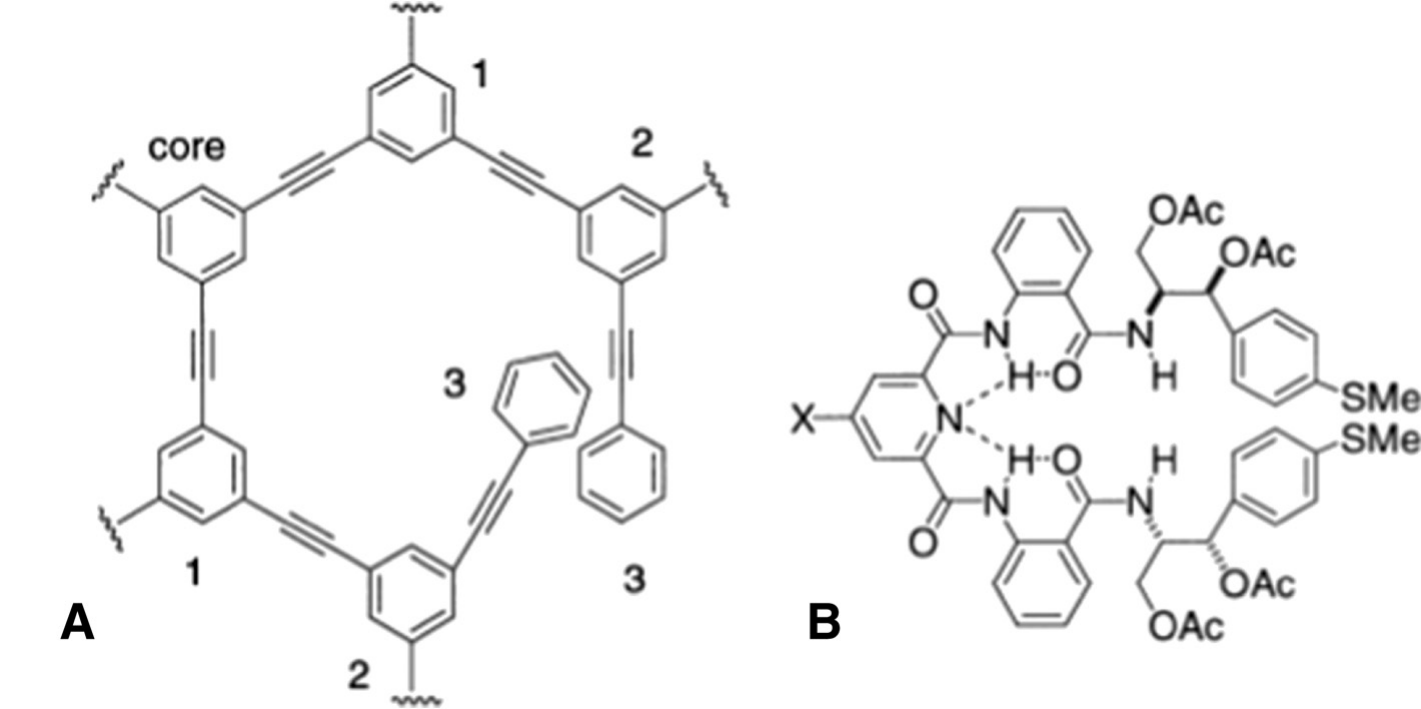
**Types of dendrimers. (A)** More type dendrimers consisting of phenyl acetylene subunits at the third-generation different arms may dwell in the same space, and the fourth-generation layer potential overlaps with the second-generation layer. **(B)** Parquette-type dendrons are chiral, non-racemic, and with intramolecular folding driven by hydrogen bonding [24].

The structure of dendrimer molecules begins with a central atom or group of atoms labeled as the core. From this central structure, the branches of other atoms called ‘dendrons’ grow through a variety of chemical reactions. There continues to be a debate about the exact structure of dendrimers, in particular whether they are fully extended with maximum density at the surface or whether the end-groups fold back into a densely packed interior [21,22]. Dendrimers can be prepared with a level of control not attainable with most linear polymers, leading to nearly monodisperse, globular macromolecules with a large number of peripheral groups as seen in Figure 2, the structure of some dendrimer repeat units, for example, the 1,3-diphenylacetylene unit developed by Moore [23].

**Figure 3:**
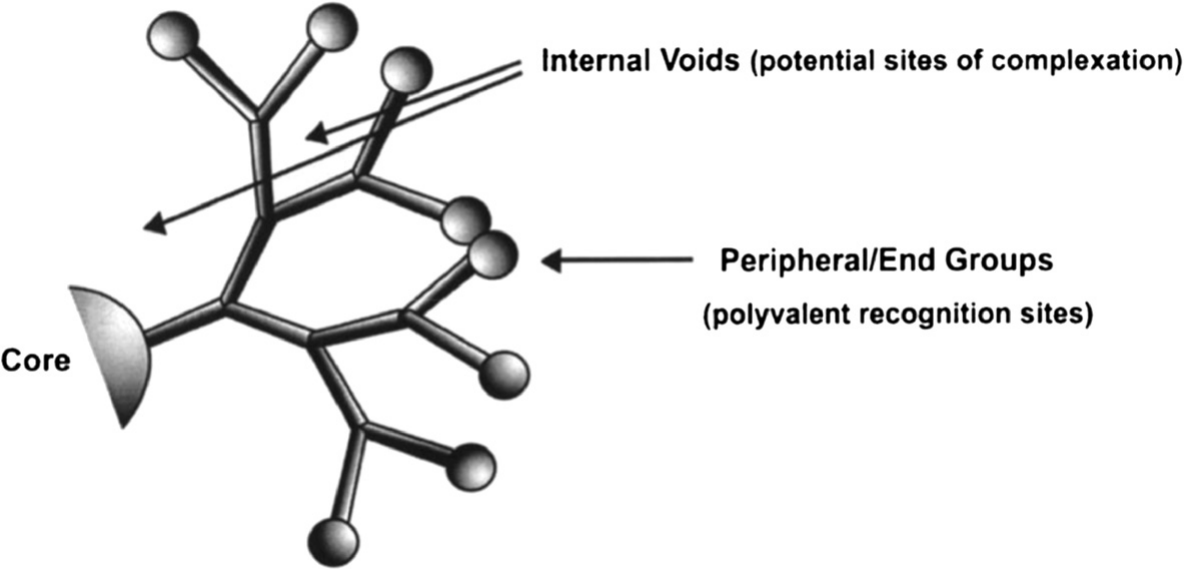
Three main parts of a dendrimer: the core, end-groups, and subunits linking the two molecules.

Dendrimers are a new class of polymeric belongings. Their chemistry is one of the most attractive and hastily growing areas of new chemistry [25-27]. Dendrimer chemistry, as other specialized research fields, has its own terms and abbreviations. Furthermore, a more brief structural nomenclature is applied to describe the different chemical events taking place at the dendrimer surface. Dendrigrafts are a class of dendritic polymers like dendrimers that can be constructed with a well-defined molecular structure, i.e., being monodisperse [28]. The unique structure of dendrimers provides special opportunities for host-guest chemistry (Figure 3) and is especially well equipped to engage in multivalent interactions. At the same time, one of the first proposed applications of dendrimers was as container compounds, wherein small substrates are bound within the internal voids of the dendrimer [29]. Experimental evidence for unimolecular micelle properties was established many years ago both in hyperbranched polymers [30] and dendrimers [31].

### Synthesis

Dendrimers are just in between molecular chemistry and polymer chemistry. They relate to the molecular chemistry world by virtue of their step-by-step controlled synthesis, and they relate to the polymer world because of their repetitive structure made of monomers [32-35]. The three traditional macromolecular architectural classes (i.e., linear, cross-linked, and branched) are broadly recognized to generate rather polydisperse products of different molecular weights. In contrast, the synthesis of dendrimers offers the chance to generate monodisperse, structure-controlled macromolecular architectures similar to those observed in biological systems [36,37]. Dendrimers are generally prepared using either a divergent method or a convergent one [38]. In the different methods, dendrimer grows outward from a multifunctional core molecule. The core molecule reacts with monomer molecules containing one reactive and two dormant groups, giving the first-generation dendrimer. Then, the new periphery of the molecule is activated for reactions with more monomers.

### Cascade reactions are the foundation of dendrimer synthesis

**Figure 4:**
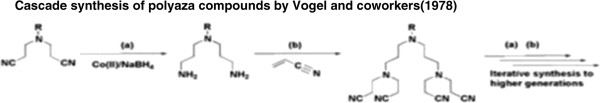
**Cascade reaction sequences developed for the synthesis of ‘non-skid-chain like’ polyazamacrocyclic compounds **[40]**.**

The basic cascade or iterative methods that are currently employed for synthesis were known to chemists much earlier. For example, similar schemes form the basis of solid-phase peptide synthesis. In turn, biology has long exploited similar iterative strategies in biochemical synthetic pathways; one example is provided by fatty acid biosynthesis [39] (Figure 4).

### The synthesis of dendrimers follows either a divergent or convergent approach

**Figure 5:**
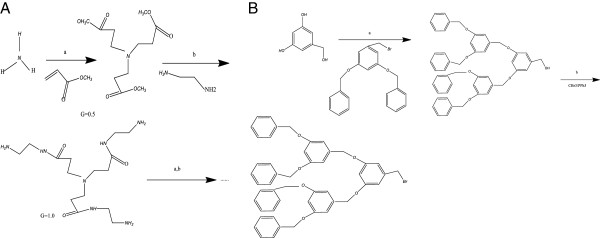
**Approaches for the synthesis if dendrimers. (A)** Divergent approach: synthesis of radially symmetric polyamidoamine (PAMAM)dendrimers using ammonia as the trivalent core; the generations are added at each synthetic cycle (two steps), leading to an exponential increase in the number of surface functional groups [37]. **(B)** Convergent approach: synthesis of dendrons or wedges or branches that will become the periphery of the dendrimer when coupled to a multivalent core in the last step of the synthesis [13].

Dendrimers can be synthesized by two major approaches. In the divergent approach, used in early periods, the synthesis starts from the core of the dendrimer to which the arms are attached by adding building blocks in an exhaustive and step-wise manner. In the convergent approach, synthesis starts from the exterior, beginning with the molecular structure that ultimately becomes the outermost arm of the final dendrimer. In this strategy, the final generation number is pre-determined, necessitating the synthesis of branches of a variety of requisite sizes beforehand for each generation [41] (Figure 5).

### Properties of dendrimers

When comparing dendrimers with other nanoscale synthetic structures (e.g., traditional polymers, buck balls, or carbon nanotubes), these are either highly non-defined or have limited structural diversity.

### Pharmacokinetic properties

Pharmacokinetic properties are one of the most significant aspects that need to be considered for the successful biomedical application of dendrimers, for instance, drug delivery, imaging, photodynamic therapy, and neutron capture therapy. The diversity of potential applications of dendrimers in medicine results in increasing interest in this area. For example, there are several modifications of dendrimers' peripheral groups which enable to obtain antibody-dendrimer, peptide-dendrimer conjugates or dendritic boxes that encapsulate guest molecules [42].

### Covalent conjugation strategies

**Figure 6:**
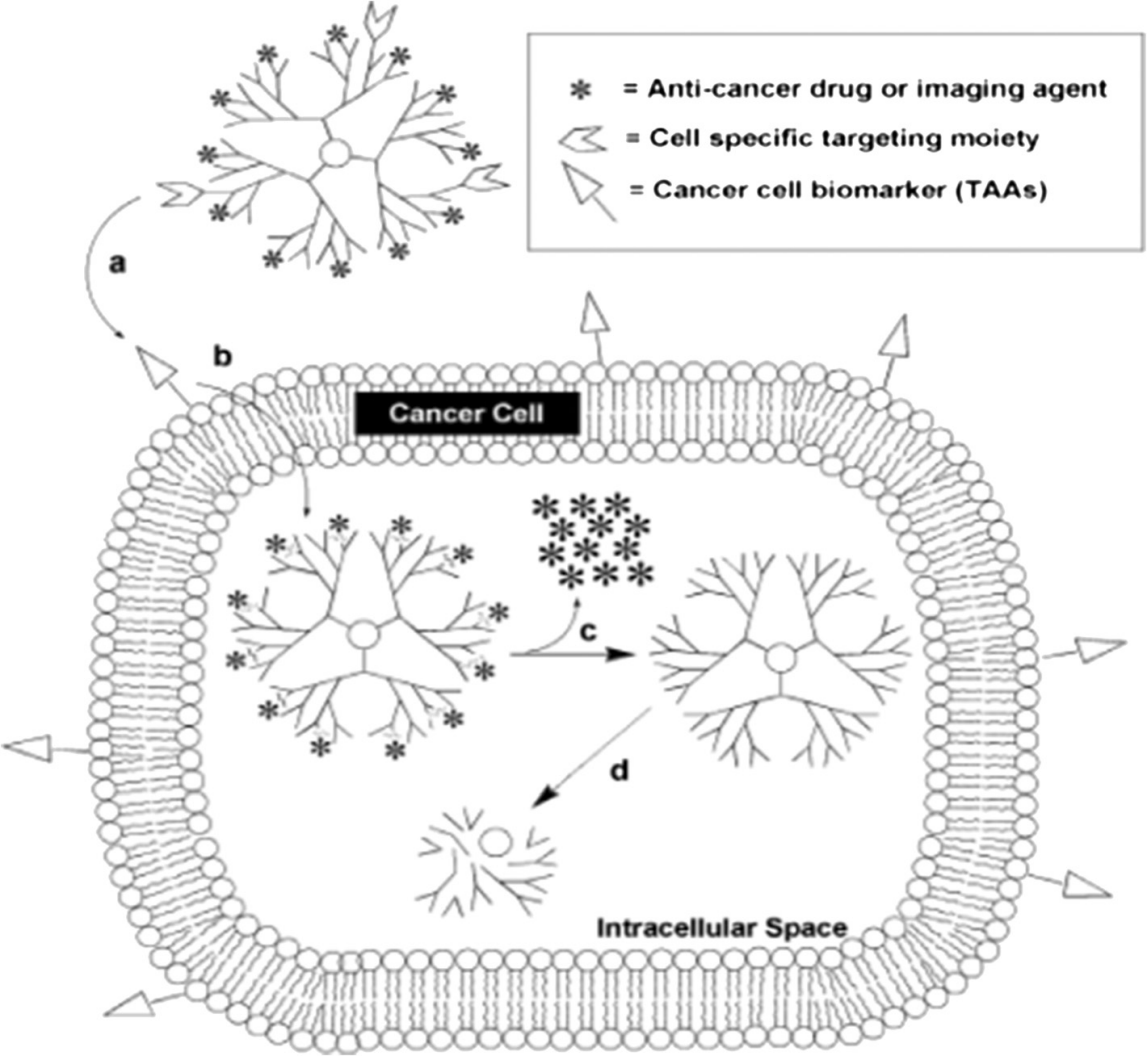
**Requirements for dendrimer-based, cancer-targeted drug delivery. (A)** Dendrimers with multiple surface functional groups can be directed to cancer cells by tumor-targeting entities that include folate or antibodies specific for tumor-associated antigens (TAAs). **(B)** The next step is ingestion into the cell which, in the case of folate targeting, occurs by membrane receptor-mediated endocytosis. **(C)** Once inside the cell, the drug generally must be released from the dendrimer, which, for the self-immolative method, results in the simultaneous disintegration of the dendritic scaffold **(D)**.

The strategy of coupling small molecules to polymeric scaffolds by covalent linkages to improve their pharmacological properties has been under experimental test for over three decades [43-46]. In most cases, however, the conjugated dendritic assembly functions as ‘pro-drug’ where, upon internalization into the target cell, the conjugate must be liberated to activate the drug (Figure 6).

### Polyvalency

Polyvalency is useful as it provides for versatile functionalization; it is also extremely important to produce multiple interactions with biological receptor sites, for example, in the design of antiviral therapeutic agents.

### Self-assembling dendrimers

**Figure 7:**
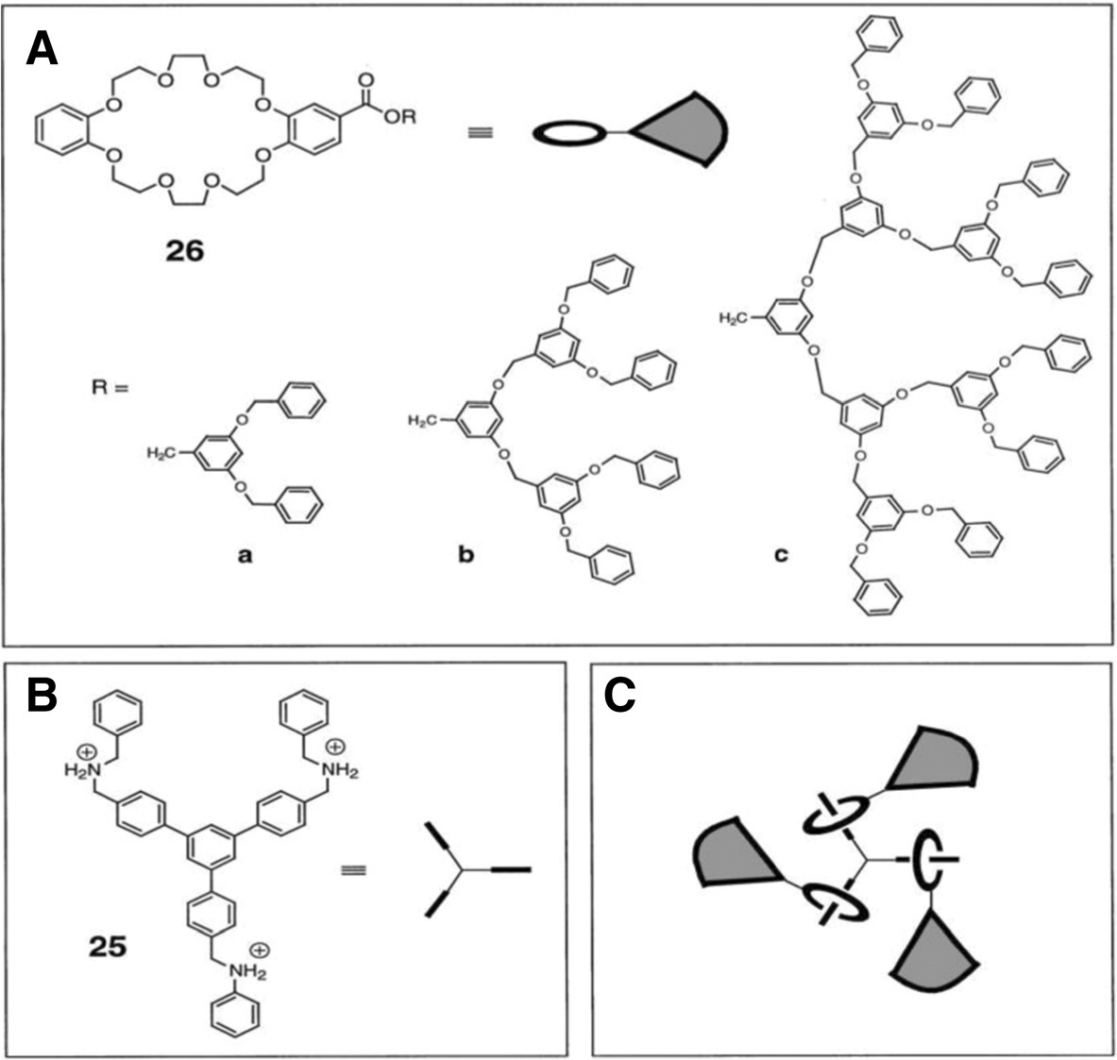
**Gibson's self-assembling dendrimers using pseudorotaxane formation. (A)** Crown ethers with dendritic substituents. **(B)** Triammonium ion core. **(C)** Schematic of tridendron formed by triple pseudorotaxane self-assembly.

Another fascinating and rapidly developing area of chemistry is that of self-assembly. Self-assembly is the spontaneous, precise association of chemical species by specific, complementary intermolecular forces. Recently, the self-assembly of dendritic structures has been of increasing interest [47]. Because dendrimers contain three distinct structural parts (the core, end-groups, and branched units connecting the core and periphery), there are three strategies for self-assembling dendrimers. The first is to create dendrons with a core unit that is capable of recognizing itself or a ditopic or polytopic core structure, therefore leading to spontaneous formation of a dendrimer [48-51]. A self-assembling dendrimer using pseudorotaxane formation as the organizing force was reported by Gibson and coworkers (Figure 7) [52].

### Electrostatic interactions

Molecular recognition events at dendrimer surfaces are distinguished by the large number of often identical end-groups presented by the dendritic host. When these groups are charged, the surface may have as a polyelectrolyte and is likely to electrostatically attract oppositely charged molecules [53]. One example of electrostatic interactions between polyelectrolyte dendrimers and charged species include the aggregation of methylene blue on the dendrimer surface and the binding of EPR probes such as copper complexes and nitroxide cation radicals [54,55].

### Applications

Today, dendrimers have several medicinal and practical applications.

#### Dendrimers in biomedical field

Dendritic polymers have advantage in biomedical applications. These dendritic polymers are analogous to protein, enzymes, and viruses, and are easily functionalized. Dendrimers and other molecules can either be attached to the periphery or can be encapsulated in their interior voids [56]. Modern medicine uses a variety of this material as potential blood substitutes, e.g., polyamidoamine dendrimers [57].

#### Anticancer drugs

Perhaps the most promising potential of dendrimers is in their possibility to perform controlled and specified drug delivery, which regards the topic of nanomedicine. One of the most fundamental problems that are set toward modern medicine is to improve pharmacokinetic properties of drugs for cancer [58]. Drugs conjugated with polymers are characterized by lengthened half-life, higher stability, water solubility, decreased immunogenicity, and antigenicity [59]. Unique pathophysiological traits of tumors such as extensive angiogenesis resulting in hypervascularization, the increased permeability of tumor vasculature, and limited lymphatic drainage enable passive targeting, and as a result, selective accumulation of macromolecules in tumor tissue. This phenomenon is known as ‘enhanced permeation and retention’ (EPR) [58,60]. The drug-dendrimer conjugates show high solubility, reduced systemic toxicity, and selective accumulation in solid tumors. Different strategies have been proposed to enclose within the dendrimer structure drug molecules, genetic materials, targeting agents, and dyes either by encapsulation, complexation, or conjugation.

#### Dendrimers in drug delivery

In 1982, Maciejewski proposed, for the first time, the utilization of these highly branched molecules as molecular containers [61]. Host-guest properties of dendritic polymers are currently under scientific investigation and have gained crucial position in the field of supramolecular chemistry. Host-guest chemistry is based on the reaction of binding of a substrate molecule (guest) to a receptor molecule (host) [62].

#### Transdermal drug delivery

Clinical use of NSAIDs is limited due to adverse reactions such as GI side effects and renal side effects when given orally. Transdermal drug delivery overcomes these bad effects and also maintains therapeutic blood level for longer period of time. Transdermal delivery suffers poor rates of transcutaneous delivery due to barrier function of the skin. Dendrimers have found applications in transdermal drug delivery systems. Generally, in bioactive drugs having hydrophobic moieties in their structure and low water solubility, dendrimers are a good choice in the field of efficient delivery system [63].

#### Gene delivery

The primary promise that the combination of understanding molecular pathways of disease and the complete human genome sequence would yield safer and more efficient medicines and revolutionize the way we treat patients has not been fulfilled to date. However, there is little doubt that genetic therapies will make a significant contribution to our therapeutic armamentarium once some of the key challenges, such as specific and efficient delivery, have been solved [64]. The ability to deliver pieces of DNA to the required parts of a cell includes many challenges. Current research is being performed to find ways to use dendrimers to traffic genes into cells without damaging or deactivating the DNA. To maintain the activity of DNA during dehydration, the dendrimer/DNA complexes were encapsulated in a water soluble polymer and then deposited on or sandwiched in functional polymer films with a fast degradation rate to mediate gene transfection. Based on this method, PAMAM dendrimer/DNA complexes were used to encapsulate functional biodegradable polymer films for substrate-mediated gene delivery. Research has shown that the fast-degrading functional polymer has great potential for localized transfection [65-67].

#### Dendrimers as magnetic resonance imaging contrast agents

Dendrimer-based metal chelates act as magnetic resonance imaging contrast agents. Dendrimers are extremely appropriate and used as image contrast media because of their properties [56].

#### Dendritic sensors

**Figure 8:**
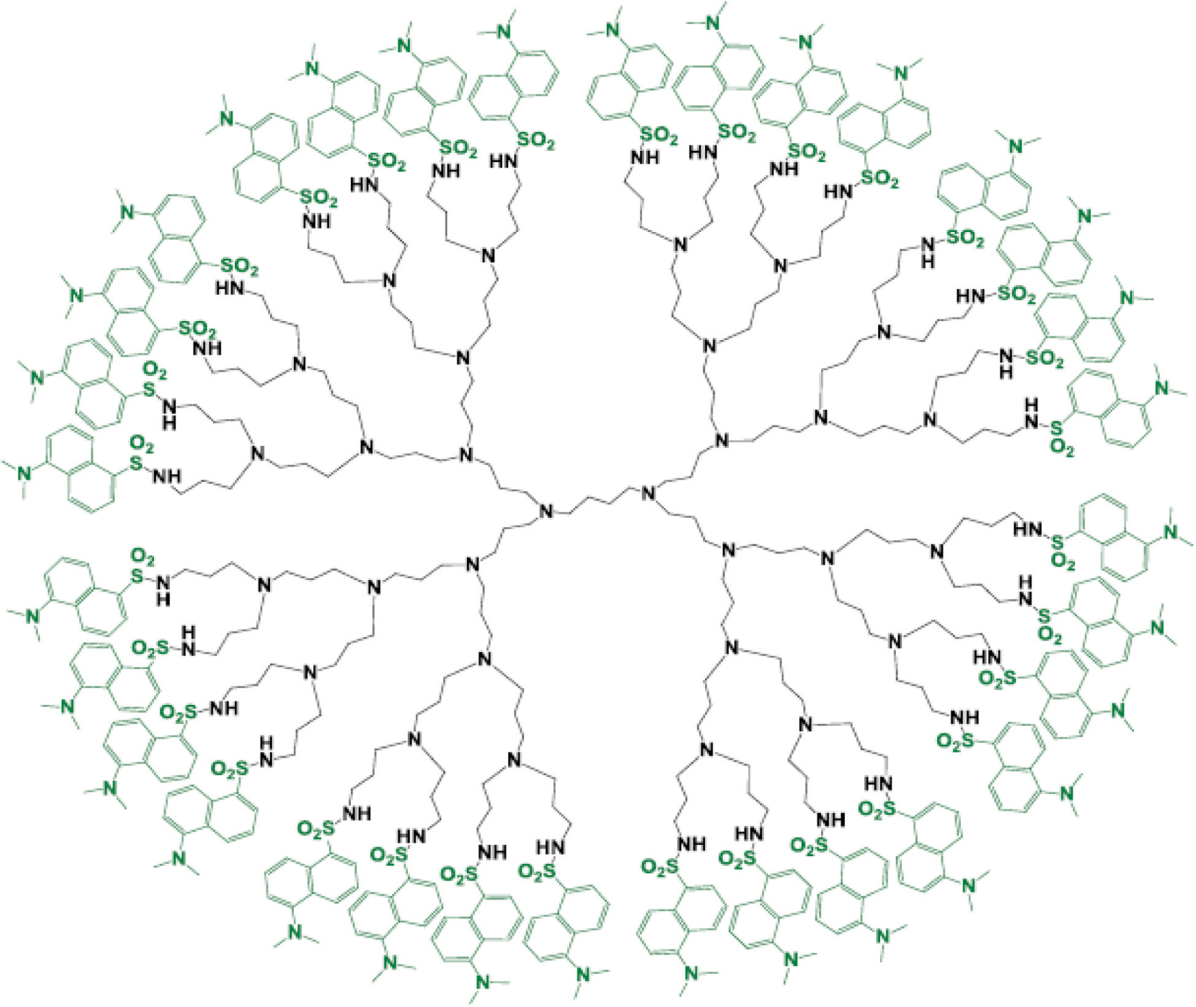
Poly (propylene amine) dendrimer, containing 32 dansyl units at its periphery.

Dendrimers, although are single molecules, can contain high numbers of functional groups on their surfaces. This makes them striking for applications where the covalent connection or close proximity of a high number of species is important. Balzani and coworkers investigated the fluorescence of a fourth-generation poly (propylene amine) dendrimer decorated with 32 dansyl units at the periphery (Figure 8) [68]. Since the dendrimer contains 30 aliphatic amine units in the interior, suitable metal ions are able to coordinate. It was observed that when a Co^2+^ ion is incorporated into the dendrimer, the strong fluorescence of all the dansyl units is quenched. Low concentrations of Co^2+^ ions (4.6 × 10^-7^ M) can be detected using a dendrimer concentration of 4.6 × 10^-6^ M. The many fluorescent groups on the surface serve to amplify the sensitivity of the dendrimer as a sensor [69].

#### Dendrimers used for enhancing solubility

PAMAM dendrimers are expected to have potential applications in enhancing solubility for drug delivery systems. Dendrimers have hydrophilic exteriors and interiors, which are responsible for its unimolecular micelle nature. Dendrimer-based carriers offer the opportunity to enhance the oral bioavailability of problematic drugs. Thus, dendrimer nano carriers offer the potential to enhance the bioavailability of drugs that are poorly soluble and/or substrates for efflux transporters [70,71].

#### Photodynamic therapy

Photodynamic therapy (PDT) relies on the activation of a photosensitizing agent with visible or near-infrared (NIR) light. Upon excitation, a highly energetic state is formed which, upon reaction with oxygen, affords a highly reactive singlet oxygen capable of inducing necrosis and apoptosis in tumor cells. Dendritic delivery of PDT agents has been investigated within the last few years in order to improve upon tumor selectivity, retention, and pharmacokinetics [72-75].

#### Miscellaneous dendrimer applications

Clearly, there are many other areas of biological chemistry where application of dendrimer systems may be helpful. Cellular delivery using carrier dendritic polymers is used in the purification of water dendrimer-based product in cosmetics contaminated by toxic metal ion and inorganic solute, and dendrimer-based commercial products organic solutes [76]. Furthermore, highly sensitive analytical devices [77,78], MRI contrast agents [79], prion research [80], burn treatment [81], and EPR imaging with spin-labeled dendrimers [82-106] are some of the diverse areas of fascinating ongoing dendrimer research that are beyond the scope of this article.

## Conclusion

Dendrimers are characterized by individual features that make them hopeful candidates for a lot of applications. Dendrimers are highly defined artificial macromolecules, which are characterized by a combination of a high number of functional groups and a compact molecular structure. A rapid increase of importance in the chemistry of dendrimers has been observed since the first dendrimers were prepared. Work was established to determine the methods of preparing and investigating the properties of the novel class of macro and micromolecules. In spite of the two decades since the finding of dendrimers, the multi-step synthesis still requires great effort.

## Competing interests

The authors declare that they have no competing interests.

## Authors' contributions

SWJ conceived the study and participated in its design and coordination. EA participated in the sequence alignment and drafted the manuscript. AA, RPA, SFA, HTN, YH, KNK, and MM helped in drafting the manuscript. All authors read and approved the final manuscript.
